# Supplementary figures and images for: Greater Durability and Protection against Herpes Simplex Viral Disease following Immunization of Mice with Single-Cycle ΔgD-2 Compared to an Adjuvanted Glycoprotein D Protein Vaccine
Source: Vaccines (Basel). 2023 Aug 14;11(8):1362. doi: 10.3390/vaccines11081362 (PMC10458853; doi:10.3390/vaccines11081362)

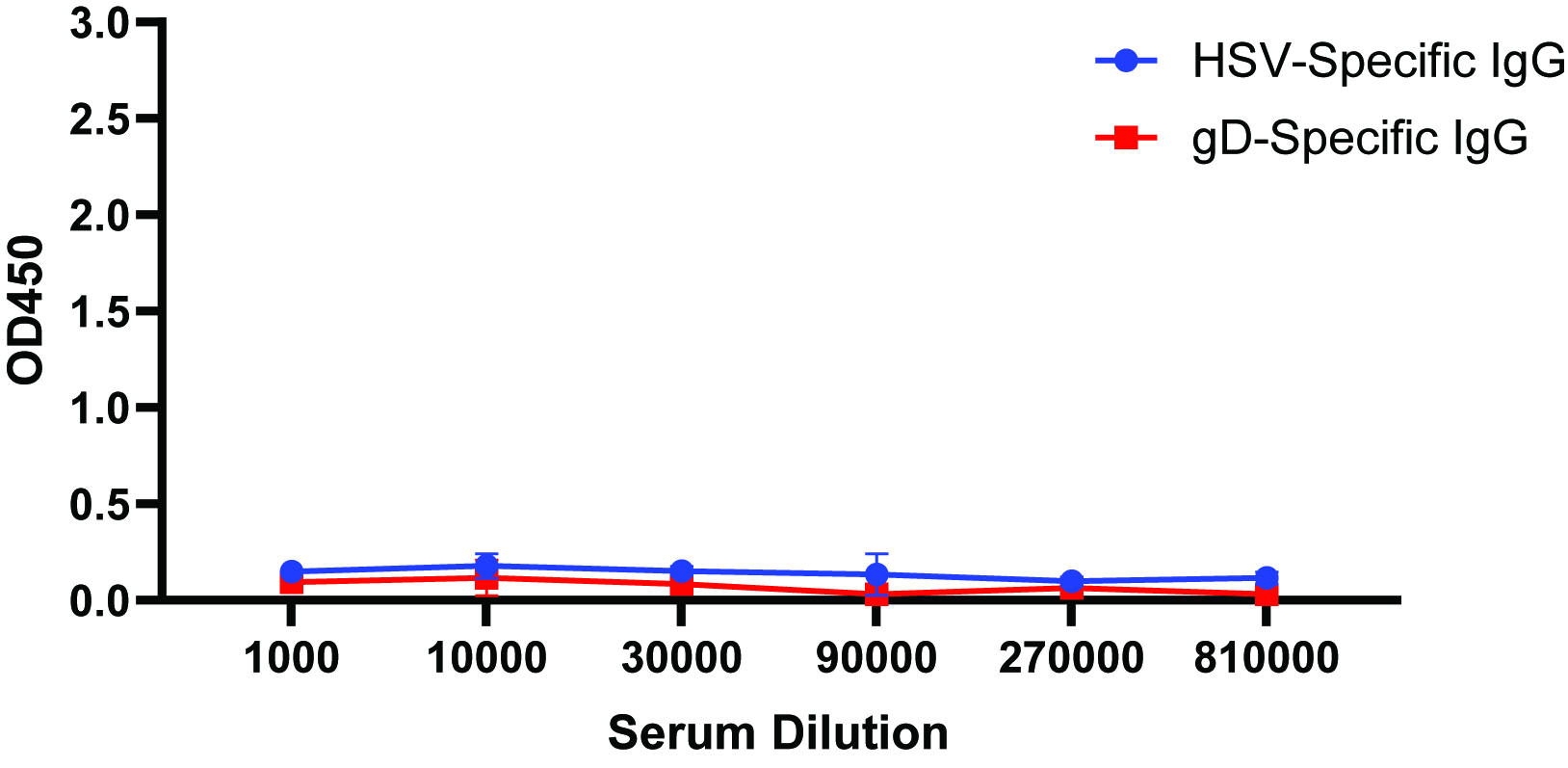

Supplement: Supplementary file 1 [file vaccines-11-01362-s001.zip › vaccines-2533306-supplementary.tif]
